# Morphology and the gradient of a symmetric potential predict gait transitions of dogs

**DOI:** 10.1007/s00422-017-0721-2

**Published:** 2017-06-19

**Authors:** Simon Wilshin, G. Clark Haynes, Jack Porteous, Daniel Koditschek, Shai Revzen, Andrew J. Spence

**Affiliations:** 1 Structure and Motion Lab, Royal Veterinary College, Hawkshead Lane, Herts, AL9 7TA UK; 2 Uber Advanced Technologies Center, 3011 Smallman St, Pittsburgh, PA 15201 USA; 3 Electrical and Systems Engineering, University of Pennsylvania, 3330 Walnut St, Philadelphia, PA 19104 USA; 4 Department of Electrical Engineering and Computer Science, University of Michigan, 1301 Beal Avenue, Ann Arbor, MI 48109 USA; 5 Bioengineering Department, Temple University, 1947 North 12th St., Philadelphia, PA 19122 USA

**Keywords:** Gaits, Dogs, Symmetry, Gait transitions, Dynamical systems

## Abstract

Gaits and gait transitions play a central role in the movement of animals. Symmetry is thought to govern the structure of the nervous system, and constrain the limb motions of quadrupeds. We quantify the symmetry of dog gaits with respect to combinations of bilateral, fore–aft, and spatio-temporal symmetry groups. We tested the ability of symmetries to model motion capture data of dogs walking, trotting and transitioning between those gaits. Fully symmetric models performed comparably to asymmetric with only a $22\%$ increase in the residual sum of squares and only one-quarter of the parameters. This required adding a spatio-temporal shift representing a lag between fore and hind limbs. Without this shift, the symmetric model residual sum of squares was $1700\%$ larger. This shift is related to (linear regression, $n=5$, $p=0.0328$) dog morphology. That this symmetry is respected throughout the gaits and transitions indicates that it generalizes outside a single gait. We propose that relative phasing of limb motions can be described by an interaction potential with a symmetric structure. This approach can be extended to the study of interaction of neurodynamic and kinematic variables, providing a system-level model that couples neuronal central pattern generator networks and mechanical models.

## Background

Examination of the dynamics of neural or mechanical aspects of moving animals has yielded important insight [1–5]. The rules that describe these dynamics are thought to remain unchanged under certain manipulations, a property termed a symmetry [6–18]. An example is left–right interchange, or bilateral symmetry. The dynamics that govern an animal running are almost identical to those of the same animal seen running in a mirror, even though the labels we would use (“left” and “right”) have been swapped.

Understanding these symmetries is important as they imply the locomotor system has a specific structure. That, in turn, means that it can be described more parsimoniously [8, 19]. Golubitsky et al. [2] employed this fact to argue for a specific structure of neuronal central pattern generator (CPG) networks based on the symmetries of quadrupedal gaits. Parsimonious descriptions of locomotion can be especially useful because it is a unifier of ultimate evolutionarily imposed constraints, neuronal control structures, body morphology, physiology and ecology.

Much of the previous evidence for these symmetries takes the form of post-diction. That is, noting that existing observations of locomotion have properties consistent with these symmetries. Here we present a theoretical framework that can predict the consequences of hypothesized symmetries and that can be tested against large, spatio-temporally rich datasets. With this tool, we directly, experimentally test previously proposed symmetries in freely moving dogs. We further extend these symmetries to describe the transitions between walking and trotting, such that a single framework can capture both gaits and the transitions between them.

Two related sets of symmetries for the dynamics governing quadrupedal locomotion have been proposed. One set relates to the nervous system [2], the other to limb movements [3]. Both have as a common hypothesis bilateral (left–right) symmetry, but the former proposes additional symmetries resulting from shifts in time, while the latter proposes the additional symmetry of fore–aft limb exchange plus time inversion.

We first test the degree to which the additional symmetries proposed by [3] are respected. Then, we extend the fore–aft plus time inversion symmetry to include a uniform phase shift and discover that this phase shift, when allowed to vary with the animal’s morphology (specifically the ratio of the leg length to the shoulder–pelvis separation), is enough to recover a good fit to experimental data.

## Methods

### Animals

A total of 5 dogs, of varying breeds, were used for this study. Dogs were volunteered and used only with the owners’ consent. The average weight of the dogs used was 23.5 kg with a range from 10.5 to 32.0 kg, and an average age of 5.4 years old, ranging from 2.5 to 7.5 years old.

### Motion capture

Kinematic data were gathered using a 12-camera Qualisys (Qualisys AB, Gothenburg, Sweden) motion capture system. A total of 28 retro-reflective markers were placed on anatomical landmarks of the dogs head, body, and legs. Data were captured in 120-second trials at 177 Hz, markers identified and disjoint tracks stitched together manually in the Qualisys Track Manager (QTM) software, and then, these data were exported to tsv files for analysis using a custom Python script.

### Experimental protocol

Dogs ran on a large equine treadmill (SÄTO I, SÄTO Treadmills, Knivsta, Sweden). From standing the speed of the treadmill was increased in 0.1–0.2 m/s increments until dogs walked comfortably. After a minimum of ten strides, the speed of the treadmill was then increased until the dogs made the transition from walk to trot. Transitions were induced by manually changing the belt speed from the characteristic speed of one gait to another with at least ten strides prior to the speed change. This was repeated, with an average of 8.2 transitions recorded per continuous bout of treadmill locomotion.

After applying our selection criteria, there were a total of 100 transitions, 55 walk-to-trot transitions, 45 trot-to-walk, with at least five transitions per dog per transition type and a minimum of 12 total transitions per dog. For some trials, the motion capture markers were occluded or otherwise poorly tracked throughout the trial. Twenty-one of these trials were excluded. This represents a potential source of systematic error, as position on a treadmill is known to affect gait [20] and occlusion is likely to occur systematically as motion capture cameras are occluded by fixtures of the treadmill. This may also systematically affect results as while this occlusion occurred for some dogs, others were successfully tracked throughout.

### Interpolation

While most markers were in view most of the time, there were occasions when individual markers would not be tracked for large segments of a stride. Marker positions were filled using a pair of Gaussian processes (a custom variant of the implementation of [21]). Gaussian process interpolation was used because interpolation with either a polynomial or a spline would not oscillate, and this would result in very poor estimates of the phase (for example, spurious jumps in the number of cycles) if a marker dropped out for a few strides.

Two Gaussian processes were used, one to remove long time-scale trends in the marker positions which trained in the normal way on the time-series data, the other filled in the short time-scale behaviour by training using phase instead of time as the independent variable.

### Leg oscillations and phase estimation

Our model predicts the time evolution of leg phases during a gait transition and can be tested against experimental measurements of these phases. Once the marker positions were interpolated, it was necessary to transform the raw marker positions on the limbs into clean, oscillatory signals centred at zero. Then, the limb phases could be calculated.

We treat each limb as a decoupled oscillator for the purpose of phase extraction, as this will cause the phase dynamics to manifest in the coupling of these oscillators.

Phase was estimated using two methods. First we used the *Phaser* algorithm [22] which combines the phase estimated by the Hilbert transform from multiple oscillatory signals and corrects them for systematics using a Fourier series. A deficiency of the *Phaser* algorithm applied to this work is that it cannot track changes in the limit cycle during transitions from walk to trot. With this as a primary motivator, we then repeated the analysis using a second method, employing a newer phase estimator, one that we refer to as “form phase”. All results shown use this second method; however, the results are qualitatively identical when using *Phaser* (all tested p values remain significant, and the patterns of the residual sum of squares remain unchanged).

A complete description of “form phase” will be submitted shortly (Wilshin et al., *in preparation*). Briefly, the “form phase” estimator works by approximating the differential form associated with the phase evolution of the dynamical system. The inner product of this differential form with the flow of the system was assumed to be constant, with this inner product being related to the average phase advance per unit time.

To compute the phases using “form phase”, we assume that the animal is constrained to a plane in state space of fixed speed when the belt speed is fixed. We then assume that within each plane of fixed speeds there is a limit cycle and a set of isochrones. Then under the assumption that the structure of the isochrones and limit cycle are slowly varying functions of speed, the limit cycle will form a distorted cylinder in the state space.

Under these assumptions, we estimate a differential one form from which we can estimate the phase by treating the speed of the dog as a extra variable in the state space with the derivative with respect to time fixed to zero. This will ensure that there is no contribution to phase advance of a limb as a result of the variation in the belt speed other than as a result of the animals reaction to the change of condition.

We begin with the full set of tracked marker positions for multiple trials with the animal walking and trotting. To remove the drift due to the motion of the body on the treadmill, the centre of mass position was subtracted from the position of all of the markers, calculating the leg coordinates in the body frame. The estimate of the centroid of the dog’s body was computed from body markers by taking an average of all body marker positions (left side of trunk, right side of trunk, base of tail, centre of back at mid-line, right pelvis, left pelvis, and withers).

We then bandpass filter these positions, combine these with their integrals and select the subset which are most sinusoidal (this was done subjectively) for each leg for each subject. We then z-score and perform PCA retaining the first two principal components which are then centred by their median and divided by their standard deviation, again for each leg and each subject. The principal components were truncated at order two as the cycle count of the third principal component did not match that of the first and second, which had large amplitude clean oscillations. By combining multiple markers, we were able to create this strong, clear oscillatory signal and ensure missing markers would only make a minor contribution to the estimated phases. This transformation process was retained and applied to the tracked positions during transitions to get a phase for each leg for each subject in each transition at each time point.

The filtered and transformed three-dimensional spatial coordinates of each leg for the training data were then used to train a phaser as noted (as described in [22]), one for each leg, and estimate the differential form. The phases of the transition trials were then estimated by applying these phase estimators to the spatial coordinates of the limbs during these trials.

Training data were identified manually with a custom MATLAB (MathWorks, Massachusetts) GUI. This tool was then further employed to identify and extract regions in which gait transitions were made.

### Modelling

We extend the model of [3] by incorporating the animal speed (*v*, the forward velocity of our dog relative to the treadmill belt). The original model has the form:

1 $$\begin{aligned} \dot{\phi }_k= & {} K_{k} +\sum _{n=1}^{\infty }A_{nk}\exp \left( in\left( \phi _k-\phi _{e_\mathrm{rl}\left( k\right) }\right) \right) \nonumber \\&+ \sum _{n=1}^{\infty }C_{nk}\exp \left( in\left( \phi _k-\phi _{e_\mathrm{fh} \left( k\right) }\right) \right) \nonumber \\&+ \sum _{n=1}^{\infty }E_{nk}\exp \left( in\left( \phi _k- \phi _{e_\mathrm{lr}\left( e_\mathrm{fh}\left( k\right) \right) }\right) \right) \nonumber \\&+ c.c. \end{aligned}$$

where the Latin indices run from 0 to 3 and 0, 1, 2 and 3 correspond to fore left, rear right, rear left and fore right, respectively, $e_\mathrm{lr}$ exchanges indices corresponding to left and right limbs, and $e_\mathrm{fh}$ exchanges indices corresponding to fore and hind, and *i* is the principle square root of $-1$ when not used as an index. The $+ c.c.$ refers to the addition of the complex conjugate of the terms on the right-hand side. The coefficients $A_{k,n}$, $C_{k,n}$ and $E_{k,n}$ are complex numbers as are the terms themselves; thus, we must add their complex conjugates to obtain real phases. The constant term, $K_k$, is excluded from this as it is assumed to be real. We note that the composition of $e_\mathrm{lr}$ and $e_\mathrm{fh}$ as seen in the last term of this equation has the effect of performing diagonal exchange of limbs. Before we can compare this model to actual transitory behaviours, it is necessary to extend it so that the parameters are a function of speed or limb cycle frequency (this model can only generate transitory behaviour between quasi-stable states). For simplicity, we will use the animals forward speed relative to the substrate (in this case the treadmill belt) and extend the model with a power series in this speed. To ease the notational burden, we introduce the phase vector:

2 $$\begin{aligned} \phi = \left( \phi _0,\phi _1,\phi _2,\phi _3\right) \end{aligned}$$

We note that operators that exchange limbs are just matrices when acting on this vector, that can be written in consultation with our limb convention, as:

3 $$\begin{aligned} E_\mathrm{LR}=\left( \begin{array}{llll} 0 &{} \quad 0 &{}\quad 0 &{}\quad 1 \\ 0 &{}\quad 0 &{}\quad 1 &{} \quad 0 \\ 0 &{}\quad 1 &{}\quad 0 &{}\quad 0 \\ 1 &{} \quad 0 &{} \quad 0 &{}\quad 0 \\ \end{array}\right) ,\quad E_\mathrm{FH}=\left( \begin{array}{llll} 0 &{} \quad 0 &{} \quad 1 &{} \quad 0 \\ 0 &{} \quad 0 &{} \quad 0 &{} \quad 1 \\ 1 &{}\quad 0 &{}\quad 0 &{}\quad 0 \\ 0 &{} \quad 1 &{} \quad 0 &{}\quad 0 \\ \end{array}\right) . \end{aligned}$$

For convenience, we define:

4 $$\begin{aligned} E_\mathrm{D}=E_\mathrm{LR}E_\mathrm{FH}=\left( \begin{array}{llll} 0 &{}\quad 1 &{}\quad 0 &{}\quad 0 \\ 1 &{}\quad 0 &{}\quad 0 &{}\quad 0 \\ 0 &{}\quad 0 &{}\quad 0 &{}\quad 1 \\ 0 &{}\quad 0 &{}\quad 1 &{}\quad 0 \\ \end{array}\right) \end{aligned}$$

which is just the diagonal interchange of limbs. With these additions, the equations governing our dynamics become:

5 $$\begin{aligned} \dot{\phi }_k= & {} \sum _{m=0}^{\infty }K_{mk}v^m \nonumber \\&+ \sum _{m=0}^{\infty }\sum _{n=1}^{\infty } A_{nmk}v^m\exp \left( in\left[ \left( I-E_\mathrm{LR}\right) \phi \right] _k\right) \nonumber \\&+\sum _{m=0}^{\infty }\sum _{n=1}^{\infty } B_{nmk}v^m\exp \left( in\left[ \left( I-E_\mathrm{FH}\right) \phi \right] _k\right) \nonumber \\&+ \sum _{m=0}^{\infty }\sum _{n=1}^{\infty } C_{nmk}v^m\exp \left( in\left[ \left( I-E_\mathrm{D}\right) \phi \right] _k\right) \nonumber \\&+ c.c. \end{aligned}$$

where *I* is the $4\times 4$ identity matrix, and the square braces with a subscript *k* denote taking the *k*th component of the vector. The $K_{mk}$, $A_{nmk}$, $B_{nmk}$ and $C_{nmk}$ are new coefficients with the same properties as the previous quantities with the same name up to an additional index for velocity. We further define the set of matrices $G=\left\{ E_\mathrm{LR},E_\mathrm{FH},E_\mathrm{D}\right\}$ allowing us to simplify our equation to:

6 $$\begin{aligned} \dot{\phi }_k= & {} \sum _{m=0}^{\infty }K_{mk}v^m \nonumber \\&+ \sum _{m=1}^{\infty }\sum _{n=1}^{\infty }\sum _{g\in G} D_{nmgk}v^m\exp \left( in\left[ \left( I-g\right) \phi \right] _k\right) \nonumber \\&+ c.c. \end{aligned}$$

where the coefficients $D_{nmgk}$ comprise the old $A_{nmk}$, $B_{nmk}$ and $C_{nmk}$ coefficients, depending on the value of *g*. We will truncate this power series to be of order one ($m=\left\{ 0,1\right\}$). Similarly, we will fix the maximum order of the Fourier series to 2 (an order less than this is insufficient to replicate the observed behaviour of quadrupeds [3]). This yields:

7 $$\begin{aligned} \dot{\phi }_k= & {} \sum _{m=0}^{1}K_{mk}v^m \nonumber \\&+ \sum _{m=0}^{1}\sum _{n=1}^{2}\sum _{g\in G} D_{nmgk}v^m \exp \left( in\left[ \left( I-g\right) \phi \right] _k\right) \nonumber \\&+ c.c. \end{aligned}$$

While several symmetry groups are considered in [3], only one, when applied to eqn. 7, is capable of supporting both walks, trots and transitions between the two:

8 $$\begin{aligned} \mathbf {C} = \big \{{\mathcal {E}},{\mathcal {O}}_\mathrm{rl},- {\mathcal {E}}{\mathcal {O}}_\mathrm{fh},-{\mathcal {E}}{\mathcal {O}}_\mathrm{fh} {\mathcal {O}}_\mathrm{rl}\big \} \end{aligned}$$

The symbols ${\mathcal {E}}$, ${\mathcal {O}}_\mathrm{rl}$, and ${\mathcal {O}}_\mathrm{fh}$ refer, respectively, to the identity, left–right interchange, and front–hind limb interchange operators. A minus sign denotes time and phase inversion. This group has four subgroups, the first is $\mathbf {C}$ itself, the others are:

9 $$\begin{aligned} \mathbf {C}_{1} = \left\{ {\mathcal {E}}\right\} \quad \mathbf {C}_\mathrm{rl} = \left\{ {\mathcal {E}},{\mathcal {O}}_\mathrm{rl}\right\} \quad \mathbf {C}_\mathrm{fh} = \left\{ {\mathcal {E}},-{\mathcal {E}} {\mathcal {O}}_\mathrm{fh}\right\} \end{aligned}$$

Each of these symmetries can be applied to the dynamics of Eq. 7 to generate a new set of equations for the animal limb phase dynamics [3].

We will illustrate how this can be reduced to a matrix transformation of the equations and use the left–right symmetry as an example. This procedure can be greatly simplified by a change of basis. We therefore re-write Eq. 6 with respect to new basis functions the 4-tuples $X_{\mu }\left( \phi \right)$:

10 $$\begin{aligned} \dot{\phi } = \sum _{\mu ,m}M_{m\mu }\circ X_{\mu }\left( \phi \right) v^m. \end{aligned}$$

where the $M_{m\mu }$ are 4-tuples of new coefficients and $\mu$ is a new index that covers both the constant term and all unique combinations of *n* and *g* (*g* an element of G, since we are truncating the series the exact ordering of the map from the old indices *n*, and *g*, does not matter) and the complex conjugates. $\circ$ is the Hadamard (elementwise) product. Applying our left–right symmetry to these equations, we find

11 $$\begin{aligned} E_\mathrm{LR}\dot{\phi } = \sum _{\mu ,m}M_{m\mu } \circ X_{\mu }\left( E_\mathrm{LR}\phi \right) v^m \end{aligned}$$

$E_\mathrm{LR}$ is just an element of a matrix representation of our group $\mathbf {C}$, and it is straightforward to calculate this representation of $\mathbf {C}$ in this space (they are the entries of Eq. 3 along with the identity, up to a change of sign). Since $E_\mathrm{LR}$ is self-inverse, we can write

12 $$\begin{aligned} \dot{\phi } = \sum _{\mu ,m}E_\mathrm{LR}M_{m\mu } \circ X_{\mu }\left( E_\mathrm{LR}\phi \right) v^m. \end{aligned}$$

We can now calculate how the left–right symmetry affects our basis. As an example, the elements of $X_\mu \left( \phi \right)$ where the matrix referred to by $\mu$ is $E_{LR}$:

13 $$\begin{aligned} \exp \left( in\left[ \left( I-E_\mathrm{LR}\right) \phi \right] _k\right) \end{aligned}$$

transforms to

14 $$\begin{aligned} \exp \left( in\left[ \left( I-E_\mathrm{LR}\right) E_\mathrm{LR}\phi \right] _k\right) =\exp \left( -in\left[ \left( I-E_\mathrm{LR}\right) \phi \right] _k\right) . \end{aligned}$$

This transformation has simply mapped one basis element to another (in this case their complex conjugate), up to a multiplicative factor. The same is true for all other basis elements under these symmetries, and so we can write our transformation on $X_{\mu }\left( \phi \right)$ as

15 $$\begin{aligned} X_{\mu }\left( E_\mathrm{LR}\phi \right) = \sum _{\nu } T_\mathrm{LR,\mu \nu }X_{\nu }\left( \phi \right) \end{aligned}$$

where the $T_{\mathrm{LR},\mu \nu }$ can be interpreted as matrices drawn from the matrix representation of the group $\mathbf {C}$, albeit in a different vector space. Our transformed equation is therefore

16 $$\begin{aligned} \dot{\phi } = \sum _{\mu ,\nu ,m}E_\mathrm{LR}M_{m\mu } \circ T_{\mathrm{LR},\mu \nu } X_{\nu }\left( \phi \right) v^m \end{aligned}$$

This can be rearranged to:

17 $$\begin{aligned} \dot{\phi } = \sum _{\mu ,\nu ,m}E_\mathrm{LR}M_{m\mu }T_{\mathrm{LR},\mu \nu } \circ X_{\nu }\left( \phi \right) v^m \end{aligned}$$

By comparing with Eq. 10, we infer that our coefficients must satisfy

18 $$\begin{aligned} M_{m\mu } = \sum _{\nu }E_\mathrm{LR}M_{m\nu }T_{\mathrm{LR},\nu \mu }. \end{aligned}$$

The same holds true for the other elements of our group $\mathbf {C}$. Although a little machinery has been introduced to reduce this process to matrix manipulations, these results are, up to the inclusion of velocity and a change of convention, identical to those obtained by [3].

Therefore to find the constraints on our coefficients, we just need to find the relevant matrix representations of the group $\mathbf {C}$ and apply these equations, which is straightforward to do in software. This software is provided in a supplementary file.

**Fig. 1:**
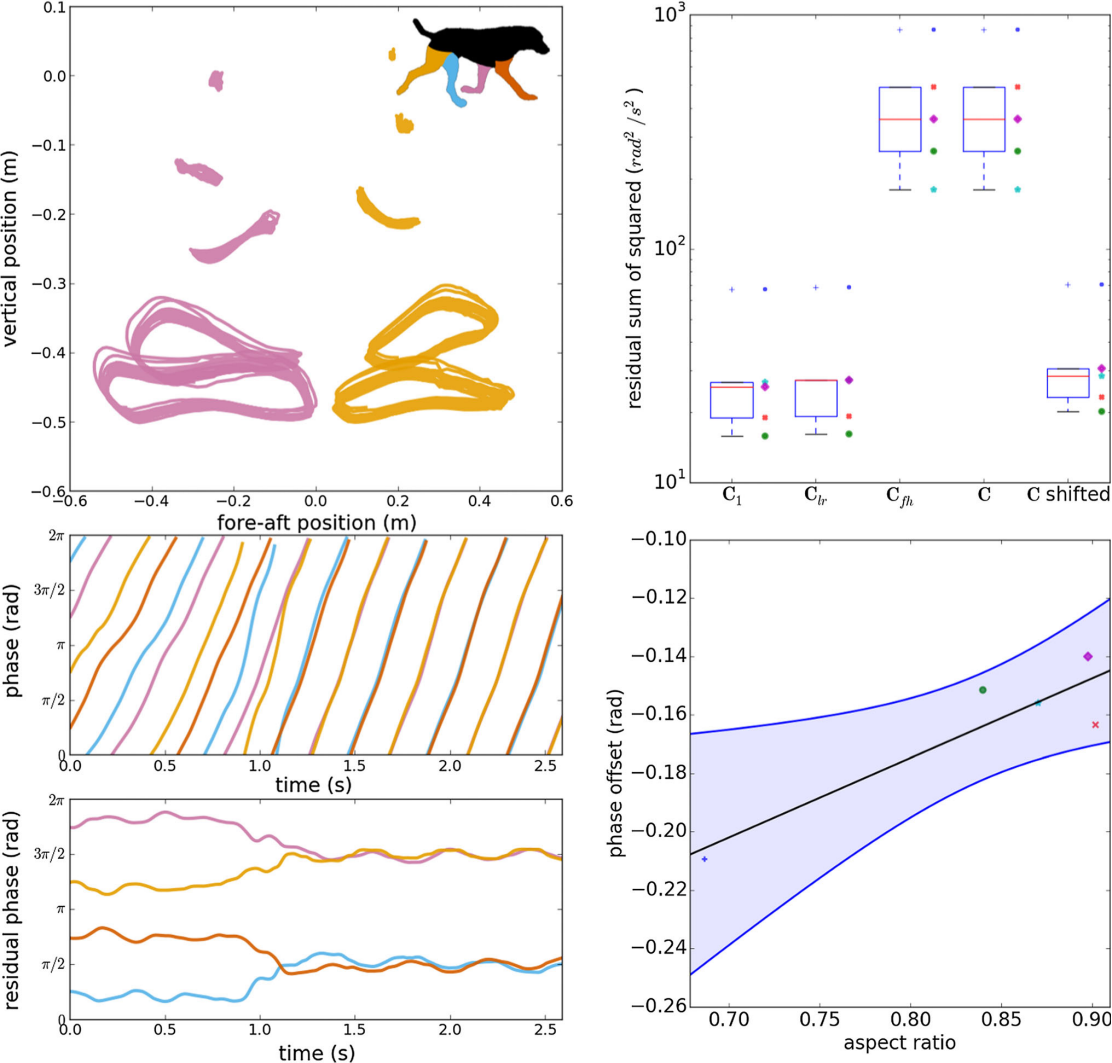
Symmetries are obeyed in moving dogs. *Left column*, estimation of animal limb state. (*top*) Centre of mass adjusted marker position from a view of the sagittal plane for two limbs (*purple* front left, *orange* rear right, *inset* indicates colour scheme for this column); (*mid*) continuous phase estimate for the limbs (*purple* and *orange* as above, *blue* rear left, *red* fore right). (*bottom*) Continuous residual phase estimate was calculated by subtracting the average phase advance from the middle figure. This example is a walk–trot transition. *Right column*, effects of symmetry. (*top*) Fit performance from residual sum of square (RSS) respecting different symmetry groups, note the y-axis is logarithmic, box plot and raster plot, each individual dog is indicated by a different marker and colour. (*bottom*) Scatter plot of morphology (labelled “aspect ratio” as this is the ratio of the fore-left limb length to the left shoulder–pelvis separation) against phase shift necessary to restore the fore–hind inversion symmetry to the dynamics of the limb motion. Blue region indicates the $95\%$ confidence bounds, each of the five dogs is indicated by a marker as above, black line is the linear line of best fit

The four subgroups here correspond to different sets of respected symmetries in animal motion, as depicted in Fig. 1. The smallest group ($\mathbf {C}_{1}$, the trivial subgroup) contains no symmetries and leaves our equations unchanged. This means that how the animals limbs will advance in phase has explicit dependence on whether the limb is a front limb or a hind limb, a left limb or a right limb.

The subgroup $\mathbf {C}_\mathrm{rl}$ implies that if we know how the left limbs couple to the other limbs, then we know how the right limbs couple, halving the number of parameters in our model. The subgroup $\mathbf {C}_\mathrm{fh}$ does the same for the fore and hind limbs up to an inversion of the dynamics in time and an inversion of phase, also halving the number of parameters. The full group assumes all of the previous symmetries apply, quartering the number of parameters.

This yields four models to compare, which we label by the corresponding symmetry groups. These models were fit to motion capture data from dogs making gait transitions on a large treadmill, computing the residual sum of squares (RSS) of a fit of each model to data.

We find that the residual sum of squares of models $\mathbf {C}$ and $\mathbf {C}_\mathrm{fh}$ are large, implying that the fore–hind symmetry is broken. We seek to correct this by the introduction of a phase shift:

19 $$\begin{aligned} (\theta _0,\theta _1,\theta _2,\theta _3) = (\phi _0,\phi _1-\Delta , \phi _2-\Delta ,\phi _3) \end{aligned}$$

As limbs 1 and 2 are the hind limbs in our convention, this amounts to applying an identical, constant phase shift to both hind limbs. One interpretation here is that the $\phi$ refer to an underlying neural oscillator, and the $\theta$ describe the limb motion, offset from the neural system by a uniform phase shift. However, further data containing measures of neural outputs would be required to determine whether this phase shift arises from the mechanical system, the neural system, or their interaction. This phase shift is applied only to the model corresponding to the largest symmetry group $\mathbf {C}$.

While the original equations proposed in [3] generate transitory behaviour in the form of quasi-static states between which the system will continuously move, they do not produce true gait transitions. Since these equations are not explicitly dependent on the limb cycle frequency or forward speed, they are incapable of having different stable gaits at different speeds/cycle frequencies.

We therefore modified them so that the model parameters depend on the animals forward velocity, thus allowing them to generate genuine transitions. It is unclear what the most appropriate control parameter is for transitions in quadrupeds. An argument for cycle frequency can be made based on the prevalence of rate encoding by the nervous system [23]. For humans, [4] presented evidence that the relevant non-specific control parameter is speed. Determining what is the most appropriate control parameter will require a study that goes beyond kinematics to probe the mechanisms of neural control.

For this study, the relation between oscillation frequency and speed is roughly monotonic. They cannot be considered independent and so the choice is less important. Any discussion herein which mentions an effect of varying “speed” can and should be viewed as an effect of speed or cycle frequency.

We need to operationalize the concept of “limb phase” if we are to model it. All phases contain an arbitrary offset, in the case of animal motion that offset is typically fixed using an event in the cycle, in our case mid-stance. It is reasonable to suppose that the symmetries we are testing are respected at the level of spinal inter-neuronal networks (CPGs), as proposed by Golubitsky et al. [2]. These networks are thought to play a critical role in locomotion by generating a basic “rhythmic” pattern, with which sensory feedback and other modulatory influences (including the mechanics of limbs and body) interact. However, our operationalization of limb phase leaves open the possibility that a phase delay has been introduced between the neural system and the limbs, which we anticipate here and subsequently address.

### Fitting to experiment

Each of the models which did not include a phase shift was fitted by computing the terms of the right-hand side of the model equation in the modelling section from the observed phases, and the terms on the left-hand side by finite differences. The resulting matrix problem is inverted to find the parameters and then the residuals computed and the residual sum of squares (RSS) of the fits calculated.

For the model with the phase shift, we again used a least squares technique, but optimize the resulting fit to reduce the RSS by adjusting $\Delta$ for each dog using a simplex optimization method [24].

### Hysteresis

Hysteresis, an overlap in the range of values of a non-specific control parameter where two qualitatively different behaviours can exist due to asymmetric transition thresholds, is an established phenomena in quadrupedal gait transitions. The walk-to-trot transition typically occurs at a higher speed than the trot-to-walk transition. We confirm that, consistent with previous observations in quadrupeds [4, 25, 26], hysteresis is present when comparing the walk-to-trot and trot-to-walk transitions.

The speeds when transitioning were estimated by fitting a sigmoid to the phase difference given by $\phi _1-\phi _0 + \phi _3-\phi _2$ (in a trot this is zero, in a walk this is *pi*) as a function of time, and the speed at the midpoint of the sigmoid estimated by nearest neighbour interpolation. The speed when transitioning from walk to trot was then compared with the speed when transitioning from trot to walk confirming the presence of hysteresis.

## Results

The RSS (in radians) for the fit of the five models to the experimental data are shown in Fig. 1.

As the results were qualitatively identical using either of the phase extraction techniques described above, we present those from the differential form based estimator, “form phase”.

Applying the left–right symmetry operator (${\mathcal {O}}_\mathrm{rl}$) to the dynamics has little effect on the RSS, implying that the left–right symmetry is respected. Both models that respect the fore–hind inversion ($-{\mathcal {E}}{\mathcal {O}}_\mathrm{fh}$) symmetry have considerably higher RSS. Often these models have bimodal residual distributions and are poor descriptions of the system.

Introducing a phase shift radically improves the fits for models which respect the fore–hind inversion symmetry and left–right symmetry. This allows a 51-parameter model to fit the data with performance comparable to the 100- or 200-parameter model. The shifts in phase for the hind limbs ($\Delta$) are seen in the bottom right panel.

We hypothesized that scaling of some aspect of the neural or mechanical systems could give rise to a size dependency in this phase shift. We therefore examined the relationship between the morphology quantified by the maximum separation of the fore-left limb toe and shoulder markers to the maximum separation of the left pelvis and shoulder markers, and this phase shift (Fig. 1). The parameter could be thought of as an “aspect ratio” of the animal. We find a statistically significant ($n=5, p=0.0328$) relationship between morphology and the phase shift. One point had considerable leverage, so the consistency of the model was checked by repeating the linear regression leaving a single data point out at a time. We confirmed that the missing point was in the $95\%$ prediction interval and that the gradient of the regression was always positive.

Hysteresis was observed: the mean ratio of the walk-to-trot transitions speeds to trot-to-walk transition speeds was 1.95. Significant differences between the walk-to-trot and trot-to-walk transition speeds were observed in all subjects ($p<0.05$).

## Discussion

We have confirmed the symmetries predicted by Schöner et al. [3] in dogs. Barring the introduction and then cancellation of asymmetric components at levels between the CPG circuitry and inter-limb coordination, it is plausible to hypothesize based on our results that the CPG circuitry will share these symmetry properties.

The origin of the morphology-dependent phase shift we have observed is important to understand if a complete picture of quadrupedal gait is to be constructed [27]. A theory of the organization of mammalian CPGs [25, 28, 29] consists of distinct rhythm generation and pattern formation layers. The delay could arise in the pattern formation layer, perhaps contributed to by transmission delays that increase with the size of the animal [30]. Alternatively, the source may be non-neuronal, such as differences in musculoskeletal properties [31], inertial or other physical properties of the body/limbs, or emergent stability mechanisms [32, 33]. These hypotheses could be differentiated by comparative study of phase shifts across scale and morphology.

Understanding these symmetries and this phase shift would allow this behaviour to be replicated in robotic systems (for example RHex [34, 35]). If these symmetries and the phase shift are adaptive, then this could correspond to an enhanced performance of these robotic systems.

Our approach makes it possible to quantify to what degree symmetries are obeyed or violated with broad applicability within movement science. Golubitsky et al. [2] proposed neural architectures for quadrupedal CPGs based on assumptions that fit with observed quadrupedal gaits and generalized this approach to myriapods. The degree to which quadrupedal locomotion agrees with the proposed neural architecture, further refinements to the structure of that architecture, and quantitative testing of the symmetries present in two-legged, six-legged, and myriapodal runners are made possible by our approach.

The symmetries presented by Schöner et al. [3] are closely related to those of Golubitsky et al. [2]. However, the former is a model of limb coordination and the latter a model of the neural substrate at the level of spinal interneurons. By introducing a phase shift between pattern generation and limb coordination, we offer a partial bridge of this gap. Our results suggest that the common symmetries these two authors propose are respected both at the neural and at the mechanical levels (subject to our modifications).

Known behaviours of quadrupedal systems are consistent with the arguments of [2, 3]. Our extension of these models to transitions along with our methodological innovations has provided further evidence that these symmetry approaches to model construction reflect something fundamental in animal locomotion and can make successful predictions in novel contexts.
